# Inflammatory macrophages in pancreatic acinar cell metaplasia and initiation of pancreatic cancer

**DOI:** 10.18632/oncoscience.151

**Published:** 2015-03-28

**Authors:** Geou-Yarh Liou, Peter Storz

**Affiliations:** ^1^ Department of Cancer Biology, Mayo Clinic Comprehensive Cancer Center, Mayo Clinic, Jacksonville, Florida, USA

## Abstract

The roles of inflammatory macrophages in pancreatic tissue and the development of pancreatic cancer have not been well characterized. Recently it was shown that inflammatory macrophages, besides their function in clearing dead cells, also initiate pancreatic acinar cell metaplasia to duct-like progenitor cells. While in pancreatitis this is a reversible process, in context of an oncogenic stimulus this process is irreversible and can lead to the formation of precancerous lesions. Recent work now indicates that acquisition of an activating Kras mutation in acinar cells initiates signaling that leads to chemoattraction of M1-poliarized macrophages. This oncogene-caused chronic microinflammation can accelerate the pathogenesis of pancreatic cancers.

## INTRODUCTION

Infiltration of macrophages into the pancreas occurs during acute or chronic pancreatitis; and chronic pancreatitis is tightly-linked to development and progression of pancreatic ductal adenocarcinoma (PDA). While the roles of macrophages in pancreatitis are somewhat well established, only little is known on their role in the development of PDA. Differently polarized macrophages (M1 or M2) can exhibit pro- and anti-inflammatory properties. M1-polarized macrophages not only remove dead or dying cells via phagocytosis, but are also involved in inflammation-mediated tissue remodeling, while M2-polarized macrophages restrain the inflammatory response and also inhibit the T-cell response [1]. Therefore, for PDA, M2-polarized macrophages have been described as the classical tumor promoting macrophages, while M1-polarized macrophages have been suggested to be tumor preventing. For pancreas biology, the view on M1 macrophages changed with recent publications showing that M1-polarized macrophages can initiate and drive acinar cell transdifferentiation to a duct-like progenitor cell type, a process called acinar-to-ductal metaplasia (ADM) [2]. These ADM progenitor cells in context of oncogenic signaling (Kras mutation or augmented EGF-R) initiate the formation of pancreatic lesions and eventually development of PDA [3].

### Inflammatory macrophages as drivers of the ADM process in pancreatitis

**Figure 1:**
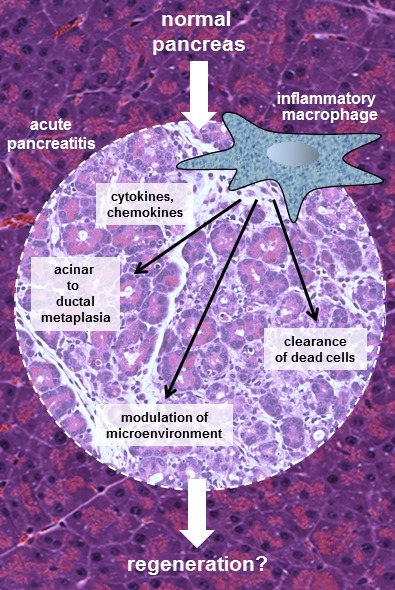
Inflammatory macrophages drive the ADM process during acute pancreatitis During acute pancreatitis acinar cells can either undergo cell death or a transdifferentiation process named acinar-to-ductal metaplasia (ADM). ADM changes their phenotype to pancreatic progenitor-like cells, which could be important for pancreatic repopulation. Inflammatory macrophages can initiate ADM through release of cytokines and chemokines. They also regulate removal of dead cells and modulation of the microenviroment. The net effect of these different functions of inflammatory macrophages possibly leads to pancreatic regeneration.

Acute pancreatitis is a sudden inflammation of the pancreas that can have multiple causes including alcohol abuse, smoking or unhealthy diet. It is driven by damage of pancreatic acinar cells and release of digestive enzymes and pro-inflammatory messengers that lead to macrophage infiltration and activation [4]. During acute pancreatitis pancreatic acinar cells can undergo apoptotic or necrotic cell death or acinar-to-ductal metaplasia. Infiltrated macrophages contribute to clearance of debris and damaged cells, stimulate a further immune response by recruiting T-cells and neutrophils; and often severe the disease to systemic inflammatory response syndrome [5]. However, macrophages also orchestrate the resolution of inflammation by producing anti-inflammatory mediators and may drive processes contributing to pancreas tissue regeneration [6]. For example, acinar-to-ductal metaplasia generates a cellular phenotype that expresses markers for pancreatic progenitor cells. Using animal models and 3D organoid culture of acinar cells, it was demonstrated that during acute pancreatitis macrophages can drive the ADM process [2]. This is mediated through macrophage-secreted cytokines such as tumor necrosis factor (TNF) and chemokines such as CCL5/RANTES [2]. In addition, inflammatory macrophages secrete proteinases such as matrix metalloproteinases (MMPs) that contribute to remodeling of the microenvironment, or induce the expression of MMPs in acinar cells that undergo ADM [2, 3, 6]. Overall the interplay between removal of dead cells and debris, alterations in the microenvironment and the generation of progenitor cells (via ADM) – all caused by inflammatory macrophages – may provide the setting for pancreas regeneration after injury (Figure 1).

While rapid production of anti-inflammatory cytokines self-limits acute pancreatitis, the failure to terminate pro-inflammatory signaling prevents regeneration and leads to chronic pancreatitis [7]. In humans and genetic animal models chronic pancreatitis clearly has been identified as a risk factor for the development of precancerous lesions and PDA [8-11]. This may be explained by the constant presence of macrophages generating an ADM cell type [2, 3], since these progenitor cells in presence of proto-oncogenic signaling (i.e. an activating Kras mutation) can progress to a pre-neoplastic cell type such as the one forming pancreatic intraepithelial neoplasia (PanIN) [12]. Another mechanism of how pancreatitis contributes to development of PDA is by inhibiting Kras-induced senescence [10].

### M1-polarized macrophages contribute to the initiation of Kras-caused pre-neoplastic lesions

**Figure 2:**
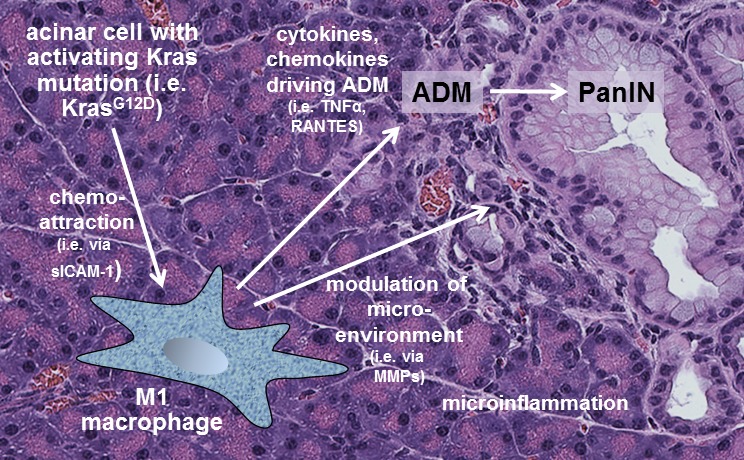
M1-polarized macrophages contribute to the initiation of pre-neoplastic lesions Acinar cells that acquire an activating Kras mutation (i.e. KrasG12D) can cause microinflammation by expressing chemoattractants for M1 macrophages. M1 macrophages then drive ADM and the formation of pre-neoplastic lesions by providing cytokines, chemokines and proteinases that modulate pancreatic microenvironment.

In order to drive the development of pancreatic cancer, oncogenic Kras needs additional inflammatory stimuli to reach pathological activity levels [13, 14]. Even more intriguing is that the acquisition of an oncogenic Kras mutation in acinar cells can initiate microinflammation and chemoattraction of M1-polarized macrophages [3]. This is mediated through induction of expression of intracellular adhesion molecule-1 (ICAM-1). ICAM-1 is a surface molecule that can be shed into a soluble form (sICAM-1) [15]. sICAM-1 serves as a chemoattractant for M1-polarized macrophages, while M2-polarized macrophages are not attracted [3]. Attracted macrophages then contribute to metaplasia and formation of pre-neoplastic lesions by secreting proteinases such as matrix-metalloproteinases (MMPs) that facilitate microenvironment remodeling, as well as tumor necrosis factor (TNF) and other inflammatory cytokines (Figure 2). The requirement of such crosstalk between acinar cells with Kras mutations and M1-polarized macrophages as a necessary event for the initiation of pancreatic precancerous lesions was demonstrated by depletion of macrophages in animals which attenuated the progression of Kras-caused lesions. Similar effects have been obtained with ICAM-1 neutralizing antibodies [3].

### Open Questions and Future Challenges

Understanding the crosstalk between the pancreatic microenvironment and acinar cells expressing oncogenic mutations is important for several reasons. First, it may lead to discovery of early markers predicting cancer development; and second it could reveal new strategies for intervention.

The finding that M1-polarized macrophages can contribute to tumor initiation is somewhat surprising, since tumor associated macrophages (TAM) often are of a M2-polarized phenotype. M1-polarized macrophages so far mainly have been seen as anti-tumorigenic. An immediate question is why M1-polarized macrophages, once attracted to regions of ADM, do not kill precancerous cells? This may be explained by the high antioxidant capacity of pancreatic cells that express an oncogenic version of Kras [16]. Macrophages induce cell death of target cells via reactive oxygen species (ROS), and mutant Kras has been shown to upregulate Nrf2, a transcription factor that upregulates a multitude of antioxidant genes [16].

It is also unclear if at some point during initiation or progression of pancreatic lesions signaling is initiated that leads to a switch from M1- to M2-polarized phenotypes [17]. M2-polarized macrophages are needed at a later stage in tumor development to facilitate immunosuppression [18] and angiogenesis [19]. They accelerate lymphatic metastasis and are generally associated with poor prognosis [20]. An open question is if there is a switch from the M1 to the M2 phenotype, or if the two groups are independently recruited.

Eventually, the understanding of the crosstalk between macrophages, pancreatic cells and the pancreatic microenvironment can be important to develop new treatment strategies. Different possibilities arise to target macrophage functions in pancreatitis or developing pancreatic cancer. First, chemoattraction could be blocked using neutralizing antibodies targeting soluble ICAM-1. We have demonstrated that such a strategy is effective in mice to inhibit formation of KrasG12D-caused PanINs [3]. Similar to our study, mouse monoclonal ICAM-1-blocking antibodies also have been sucessfully-tested in several other animal studies for different disease models *in vivo* [21, 22].

Second, macrophages could be directly targeted. In animal studies, macrophages can be inhibited experimentally with gadolinium chloride [2], liposome encapsulated dichloromethylene-diphosphonate [23], PAF antagonists [24], or macrophage pacifying compounds [25, 26] to prevent pancreatitis. In the developing cancer, ablation of macrophages using gadolinium chloride also has been demonstrated to prevent KrasG12D-mediated microinflammation and formation of precancerous lesions [3]. Other possibilities to deplete macrophage populations in the developing tumor are to block differentiation of hematopoietic stem cells into macrophages by targeting colony stimulating factor 1 (CSF1) or its receptor using chemical compounds or blocking antibodies. In addition, CD40 agonists destruct the tumor stroma by targeting macrophages and also reestablish the tumor immune surveillance in PDA [27].

Macrophages show high plasticity and can change their phenotype and physiology dependent on the microenvironment. Therefore, a third possibility for intervention is to modulate macrophage phenotypes. For example preventing a M1 to M2 subtype switch in pancreata with KrasG12D-caused lesions, although it may increase ADM events, may also prevent progression to PanINs and pancreatic cancer. A conversion from M1 to M2 can be achieved *in vitro* after treatment with IL-4, IL-10 or IL-13 [28], but it is unclear if this can be achieved *in vivo* in a pancreas [29].

In summary, to target macrophages or macrophage populations in the developing pancreatic cancer for therapeutic applications requires a full understanding of the phenotypes involved, how they crosstalk with other pancreatic or infiltrated cells, and how this crosstalk contributes to the formation and progression of pre-neoplastic lesions.
